# Whole-Genome Sequencing of Pasteurella multocida Strain Pm1, Isolated from a Calf

**DOI:** 10.1128/mra.00042-22

**Published:** 2022-03-28

**Authors:** Satoshi Gondaira, Jumpei Fujiki, Yuki Hirano, Ryo Murata, Ikuo Uchida, Masaru Usui, Tomohito Iwasaki, Tamaki Okabayashi, Hidetomo Iwano, Hidetoshi Higuchi

**Affiliations:** a Laboratory of Animal Health, School of Veterinary Medicine, Rakuno Gakuen University, Ebetsu, Japan; b Laboratory of Veterinary Biochemistry, School of Veterinary Medicine, Rakuno Gakuen University, Ebetsu, Japan; c Animal Research Center, Agricultural Research Department, Hokkaido Research Organization, Shintoku, Japan; d Laboratory of Veterinary Bacteriology, School of Veterinary Medicine, Rakuno Gakuen University, Ebetsu, Japan; e Laboratory of Food Microbiology and Food Safety, School of Veterinary Medicine, Rakuno Gakuen University, Ebetsu, Japan; f Laboratory of Applied Biochemistry, College of Agriculture, Food and Environment Sciences, Rakuno Gakuen University, Ebetsu, Japan; g Center for Animal Disease Control, University of Miyazaki, Miyazaki, Japan; Loyola University Chicago

## Abstract

Bovine pneumonia is a disease that causes significant economic losses in livestock industries and is vital for animal welfare. The whole-genome sequence of Pasteurella multocida strain Pm1, isolated from a calf suffering from pneumonia in Japan, is reported here.

## ANNOUNCEMENT

Pasteurella multocida, a Gram-negative bacterium, is pathogenic to many host animals, including humans (1). In the livestock industry, P. multocida is a pathogen that causes pneumonia in cattle, especially in calves, leading to significant economic losses and animal welfare concerns (2). It is considered a causative agent of the bovine respiratory disease complex and is a commensal bacterium (3). Serotype classification is mainly used for classifying this bacterium, and the most commonly isolated type is serotype A. Recently, multilocus sequencing typing (MLST) has also been used for classification of P. multocida (4).

P. multocida was isolated in Japan in 2017 by culturing the nasal swabs of calves suffering from pneumonia on sheep blood agar medium (Nissui, Tokyo, Japan) at 37°C for 24 h. A single colony was isolated and cultured in Bacto brain heart infusion (Becton Dickinson Japan, Tokyo, Japan) medium at 37°C for 24 h. Bacterial cells were harvested by centrifugation at 7,000 × *g* for 20 min at 4°C and stored at −80°C until use. Genomic DNA was extracted using a Quick-DNA HMW MagBead kit (Zymo Research, Irvine, CA). The quality of the genomic DNA was evaluated based on the absorbance value measured using BioSpec-nano (Shimadzu, Kyoto, Japan), and the ratio of A_260_ to A_280_ was 1.8. The quality of the genomic DNA was evaluated using a BioTek Synergy LX multimode reader (Agilent, Santa Clara, CA) and QuantiFluor double-stranded DNA (dsDNA) system (Promega, Madison, WI).

Genomic DNA libraries for short reads were prepared using the MGIEasy FS DNA library prep set (MGI Tech, Shenzhen, China) and MGIEasy DNA Adapters-96 kit (MGI Tech) by following the instructions written in the manual. The quality of the DNA libraries was evaluated using an Invitrogen Qubit 3.0 fluorometer (Thermo Fisher, Waltham, MA), Qubit dsDNA HS kit (Thermo Fisher), fragment analyzer (Agilent), and dsDNA 915 reagent kit (Agilent). Circularized DNA was manually prepared using the MGIEasy circularization kit (MGI Tech), and DNA nanoball (DNB) was manually prepared using the DNBSEQ-G400RS high-throughput sequencing set. Genome sequences were obtained from short reads by the DNBSEQ-G400 platform using 2 × 200-bp paired-end reads. The quality of the DNA libraries for long reads was evaluated using an Invitrogen Qubit 3.0 fluorometer (Thermo Fisher) and Qubit dsDNA HS kit (Thermo Fisher). Long-read DNA libraries were prepared using rapid barcoding sequencing kit SQK-RBK004 (Oxford Nanopore Technologies, Oxford, UK) and were sequenced using a MinION R10.4 flow cell by Nanopore DNA sequencing with base calling provided by MinKNOW software v20.10. The raw reads were analyzed on the Galaxy platform (https://usegalaxy.org/) (5) using default settings in all software packages. The adapter sequences were trimmed using Cutadapt (Galaxy v3.5+galaxy1) (6) for short reads or Porechop (Galaxy v0.2.4) (7) for long reads; low-quality reads were removed using Trimmomatic (Galaxy v0.38.0) (8), quality was controlled using FastQC (Galaxy v0.72+galaxy1) (9), and hybrid *de novo* assembly was performed using Unicycler (Galaxy v0.4.8.0) (10). The assembly metrics were calculated using QUAST (Galaxy v5.0.2+galaxy3) (11). A total of 9,700,015 DNBSEQ-G400 raw reads were obtained, which included 1,527,571,798 bp, with a coverage of 582.7×. A total of 14,320 MinION raw reads were obtained, which included an *N*_50_ of 16,357 bp, average read length of 8455.5 bp, and coverage of 46.2×. The resulting assembly had a complete genome size of 2,621,560 bp and a GC content of 40.3%. The bacterial strain was identified using the Type (Strain) Genome Server (TYGS) (12). Annotations were conducted using DFAST v1.4.0 (13). Default parameters were used for all software. The obtained genome sequence included 2,566 DNA coding sequences (CDS), 19 rRNA genes, and 57 tRNA genes.

### Data availability.

The complete genome sequence of P. multocida strain Pm1 was deposited in DDBJ/ENA/GenBank under accession number AP025519. The raw reads are available in the Sequence Read Archive (SRA) database under BioProject accession number PRJDB12814 and SRA accession numbers DRX326281 (MinION) and DRX326280 (DNBSEQ-G400).
